# Supplementary material for: Development of a Multiplex Polymerase Chain Reaction-Based DNA Lateral Flow Assay as a Point-of-Care Diagnostic for Fast and Simultaneous Detection of MRSA and Vancomycin Resistance in Bacteremia
Source: Diagnostics (Basel). 2022 Nov 4;12(11):2691. doi: 10.3390/diagnostics12112691 (PMC9689860; doi:10.3390/diagnostics12112691)
Supplement: Supplementary file 1 [file diagnostics-12-02691-s001.zip › Figure S1.pdf]

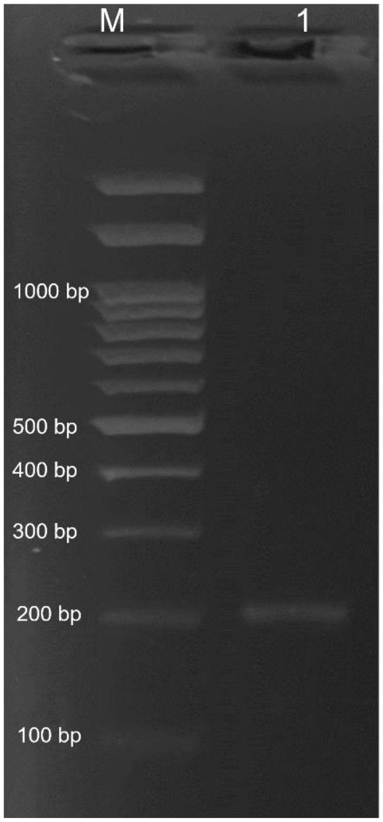
M
1
1000 bp
500 bp
400 bp
300 bp
200 bp
100 bp

A

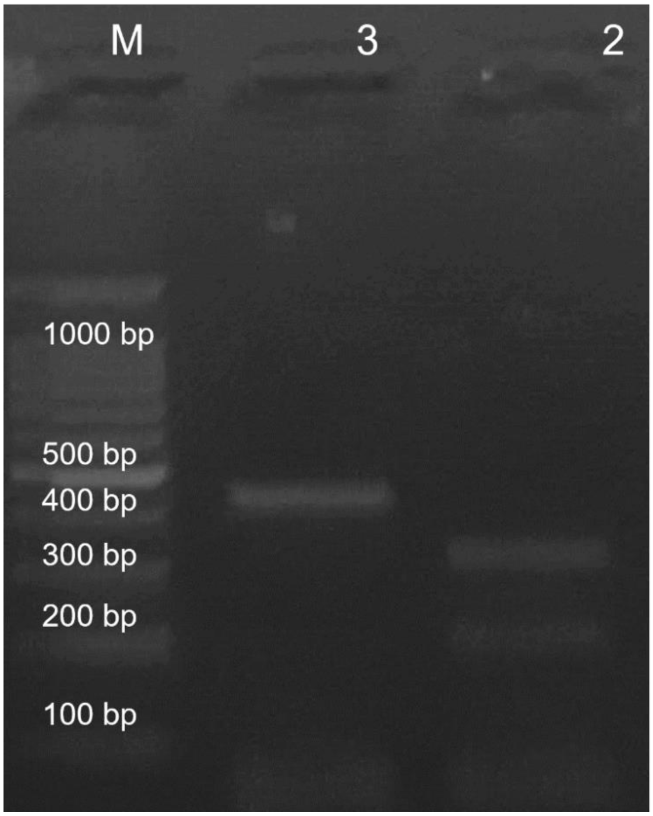
M
3
2
1000 bp
500 bp
400 bp
300 bp
200 bp
100 bp

B

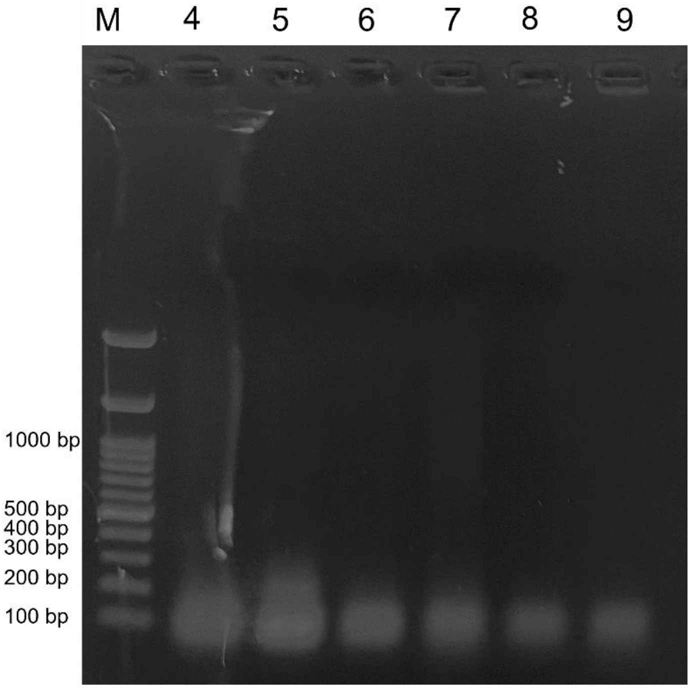
M
4
5
6
7
8
9
1000 bp
500 bp
400 bp
300 bp
200 bp
100 bp

C

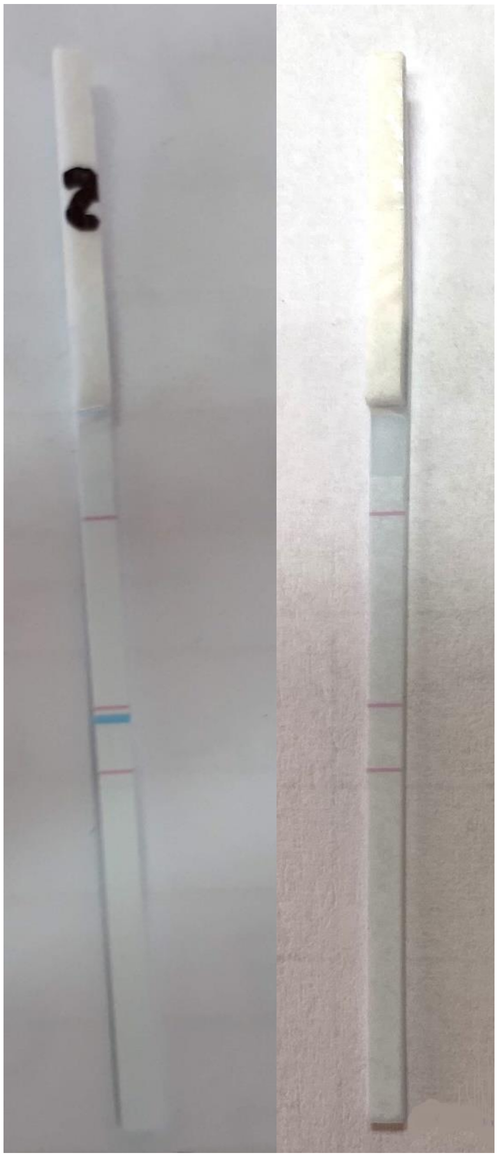

D E

Figure S1: The developed assay is specific and successfully detected (A) *Staphylococcus aureus* ATCC 25923 with one single band of 192 bp, (B) methicillin resistant *S. aureus* strain S43 with two bands of 192 bp and 310 bp and *Enterococcus faecalis* strain Y3 with a single band of 420 bp, and (C) No PCR amplification products were detectable, on agarose gel when testing other pathogens. Also, the developed multiplex polymerase reaction-based DNA lateral flow assay was specific and successfully detected (D) *nuc* gene from the genomic DNA extracted from *S. aureus* ATCC 25923 and no products were detectable when the assay was applied to other Gram-positive and Gram-negative pathogens as indicated in (E) where the assay was performed on Genomic DNA extracted from *Escherichia coli* ATCC 25922. M: is a 100 bp DNA ladder, 1: *S. aureus* ATCC 25923, 2: methicillin resistant *S. aureus* strain S43, 3: *E. faecalis* strain Y3, 4: *Acinetobacter baumannii* ATCC 19606, 5: *E. coli* ATCC 25922, 6: *Enterococcus faecium* ATCC 27270, 7: *E. faecalis* ATCC 19433, 8: *Klebsiella pneumoniae* ATCC 10031, and 9: *Pseudomonas aeruginosa* ATCC 27856.
