# Supplementary material for: Development of a Multiplex Polymerase Chain Reaction-Based DNA Lateral Flow Assay as a Point-of-Care Diagnostic for Fast and Simultaneous Detection of MRSA and Vancomycin Resistance in Bacteremia
Source: Diagnostics (Basel). 2022 Nov 4;12(11):2691. doi: 10.3390/diagnostics12112691 (PMC9689860; doi:10.3390/diagnostics12112691)
Supplement: Supplementary file 1 [file diagnostics-12-02691-s001.zip › Table S1.pdf]

Supplementary Table S1: List of accession numbers of genomes used for primer design with the location of genes used

genome accession number	Gene name	Gene location
NBCP01000028.1	vanB	6243-7276
MG592387.1	vanA	6738-7601
NZ_CP012594.1	vanA	Complement (33760-32728)
NZ_AHBU01000043.1	vanA	Complement (5833-4802)
LJOB01000059.1	vanB	4414-5445
NC_005054.1	vanA	34299-35330
KK240934.2	vanB	Complement (33756-32725)
AE017171.1	vanA	34299-35330
NZ_JXZV02000002.1	vanA	Complement (46128-45097)
NZ_AHBT01000037	vanA	Complement (22092-21061)
NZ_AHBS01000053.1	vanA	Complement (7989-6958)
NZ_AHBR01000049.1	vanA	Complement (9147-8116)
NZ_AHBQ01000050.1	vanA	Complement (6103-5072)
NZ_AHBP01000061.1	vanA	Complement (5122-4091)
NZ_AHBO01000060.1	vanA	13688-14719
NZ_AHBN01000064.1	vanA	Complement (33421-32390)
NZ_AHBK01000092.1	vanA	4162-5193
NZ_JYAP02000002.1	vanB	Complement (11699-10668)
NZ_AHBM01000055.1	vanA	Complement (11232-10201)
NZ_AHBL01000065.1	vanA	Complement (6965-5934)
NBCP01000028.1	vanB	6243-7276
CP012594.1	vanB	Complement (33760-32728)
CP093933.1	nuc	829846–830532
CP093935.1	nuc	833486-834172
CP093930.1	nuc	830359-831045
CP092538.1	nuc	848755-849441
CP092542.1	nuc	822426-823112
CP092547.1	nuc	849115-849801
CP092552.1	nuc	845887-846573
CP092554.1	nuc	848755-849441
CP092556.1	nuc	848711-849397
CP092561.1	nuc	848244-848930
CP062314.1	mecA	2066079-2068088
CP093935.1	mecA	2027461-2029470
CP080222.1	mecA	47131-49140
CP086215.1	mecA	2067249-2069258
CP086121.1	mecA	309878-311887
CP076358.1	mecA	2003741-2005750
CP076359.1	mecA	1950293-1952302
CP092825.1	mecA	54163-56172
CP062448.1	mecA	1984161-1986170

CP062408.1	mecA	2007195-2009204
CP062366.1	mecA	2118218-2120227
